# A Case of Stone in the Bladder, at Astoria, Oregon

**Published:** 1852-12

**Authors:** Israel Moses

**Affiliations:** Surgeon, U. S. A.


ART. II.—*A Case of Stone in the Bladder, at Astoria, Oregon.* By ISRAEL MOSES, M. D., Surgeon, U. S. A.

George Washington, a colored man, aged about thirty-three years, born in Virginia, and brought up in Missouri, applied to me for medical advice, February 15, 1851, at Astoria, Oregon Territory, and gave the following account of himself: From his earliest recollections he has been troubled with symptoms of urinary disease, and has been told by his friends that they began to show themselves at two years of age. He suffered at intervals with acute stinging pain, running from the neck of the bladder to the head of the penis, his water being, at these times, of a greenish yellow tinge, and feeling, as it passed from him, as if mixed with sand ; but he never could see any, and had no opportunity of examining satisfactorily, from the fact of making water out of doors. During the paroxysms "a sudden motion of the leg would relieve him by shoving something back." During the intervals he enjoyed good health, and was able to work as well as anybody. At the age of twenty-one he was attacked with severe pain about the neck of the bladder with a sensation of something rough, running backward and forward along the urethra, which continued ten days, and was relieved by parsley and water-melon seed tea. From this time he continued well, and was able to work as a field-hand, and thought himself entirely well until about two years ago, "when he had a bad spell," which lasted sixteen or eighteen days.

He started for Oregon on the 6th of May, 1850, walked and drove a team nearly all the way, (upwards of two thousand miles !) arrived on the 30th of August, and continued well a month, when the former symptoms returned ; but he worked until the middle of October, when he was obliged to give up. He was also attacked with diarrhea, which continued until the latter part of January. During these *paroxysms*, after passing a little urine, the stream would suddenly stop, and he felt a desire to strain, "but this only made the matter worse, and he was obliged to ease off," so as to evacuate the bladder by drops. He has never been examined by any surgeon, but various physicians have told him, "it could not be stone on account of its coming on him at so early an age." He is now in good general health, complaining only of a tendency to diarrhea. Upon examination with a steel sound, I soon confirmed the diagnosis formed from the rational symptoms: the instrument struck a stone as soon as it reached the bladder. I placed him in my hospital upon a preparatory course for lithotomy.

March 10. Has been very quiet since his entrance (on the first,) with little or no pain, until yesterday, when he had a paroxysm of suffering. His diet has been rice, tea, toast, &c; his bowels kept regular; has slept well, but been obliged to get up two or three times during the night to pass his water. To-day he has a great deal of pain at the neck of the bladder and end of the penis. Directed flaxseed tea, and two grains of opium three times a day.

16th. The patient's general health is excellent; but he has suffered for two or three days from increased pain, and I find him this morning walking up and down the ward with his penis in hands, in great agony. Having obtained a case of instruments only the day before, I appointed 1 o'clock, P. M., for the operation, and directed an enema to be administered, which operated freely before the time.

With the assistance of Dr. Tranchard, Lieut. Talbot, U. S. A., and George Gibbs, Esq, I proceeded to operate. Having placed the man in the usual position, and introduced the grooved staff, which was accomplished with difficulty from the restlessness of the patient, and his endeavors to free himself from his bonds. I had provided chloroform, and began to administer it as I introduced the sound, but it was some minutes before he was fully under its influence. Having struck the stone, so as to convince every one of its presence, I made the external (bilateral) incision, at which he wriggled a little, but soon became perfectly quiet, and I cut down to the staff and into the bladder, using a probe-pointed straight bistoury. Introducing my fore-finger and the forceps, grasped the stone, which proved to be much larger than I had anticipated. After working gently, I increased the extractive efforts, but found it impossible to bring away the stone without a larger incision. I accordingly withdrew the forceps, and introducing the bistoury along my finger, enlarged the opening in the bladder. Still I found it impossible to extract it. Having seized the stone, however, in a firm grip, and brought it as near the opening as I could, I cautiously nicked away, until I succeeded in its extraction. During the operation, which lasted about ten minutes, the patient remained insensible, occasionally making an effort to free himself, but again becoming perfectly quiet. Dr. Tranchard continued to administer chloroform, and to watch his pulse during the operation. The other gentlemen afforded me their useful assistance.

About ℥xx blood were lost from the division of a small artery. His pulse continued full and regular. As soon as the calculus was brought away, the chloroform was discontinued, his face dashed with cold water and aqua amonia held to his nose—he soon revived, began to sob and talk wildly, but soon became quite sensible. A female catheter was introduced into the bladder, through the wound, and twenty drops Magendie's Sol. Morphine administered. I visited him an hour after, and found he had lost ℥ viii or x of blood; he was restless and just seemed conscious that the operation had been performed; he shook hands and thanked me; complained of some pain in his penis. ℞. Repeat the morpine.

At 6 P. M., the bleeding still continued, losing ℥ viij since my last visit; has slept some, and been easy, but begins to be restless; moans and complains of pain in his penis and in his back, from lying in one position. ℞. Sulph. Morphia gr. x. At 7, P. M., free from pain, and has slept considerably; lost about ℥iv of blood, but no oozing now; pulse 80; skin cool and comfortable. 10, P. M.

Has slept most of the time and continues free from pain; some dropping of blood; skin warm and soft; pulse 90, good. Gave 15 drops morphine. A little after midnight, found him dozing and quiet; free from pain; slight oozing of blood, mixed with urine, through the catheter. I directed the Hospital Steward, who sat up with him, to give 10 drops morphine every two hours, so as to keep him fully under its influence.

17th. Found him very comfortable; had passed a good night, and is free from pain; no bleeding; urine flowing constantly through the catheter. I had him washed and dressed, and put in a comfortable bed. He tells me that he was *perfectly unconscious* of what was going on during the operation; he was sensible of the sound being passed into the bladder and striking the stone, but felt no pain afterwards, though 'he seemed to be in a kind of misery,' as he expresses himself, during the time he was under the influence of the anæsthetic.

18th. Slept well during the early part of the night; took 20 drops of morphine, which composed him, though he says he did not sleep much. Complains only of pain in his back and side; tongue clean and moist; skin cool; pulse 72, natural; passed some water through his penis this morning.

19th. Doing well; not a bad symptom, and no complaint of any pain; the wound looks healthy, and is filling up. I removed the catheter.

20th. Complains of pain along his right thigh and leg; general condition good; has some uneasiness in his bowels, not having had any dejection since the operation; skin warm; pulse 100; has passed some urine through the penis, but mostly from the wound. ℞. Castor Oil, with peppermint water.

21st. Complained last evening with pain and uneasiness about the bowels, and some tenderness on pressure. As the oil had not operated I repeated the dose, which operated three times, and he slept well during the remainder of the night. This morning he is groaning a good deal and complaining of pain in his right hip and leg. Has passed no urine per urethra. I introduced a gum elastic catheter, but it did not enter the bladder, the passage appearing to be plugged up by coagulated blood and lymph, which filled the eye of the catheter; his general condition is excellent; the wound looks healthy, and is almost closed.

22d. Complained yesterday of a severe headache, and about half-past five P. M., was seized with a severe rigor; during which, however, his skin was comfortably warm and perspiring; pulse 100, rather small, followed by hot and dry skin, frequent pulse, coated and sticky tongue. At the onset of the chill, he took 20 drops morphine. During the day he passed his water freely through the penis, in a stream. ℞. Pil Mass. Hydrarg., gr. x, at night.

23d. Is quite comfortable this morning; took castor oil, ℥ss, which operated twice or three times; is free from pain; has passed very little urine per urethra; introduced a catheter and drew off ℥viii, with a good deal of pus mixed with it. Complains of some pain in his right iliac region; no tenderness on pressure; pulse 84, natural; tongue clean and moist; wound healthy in appearance.

26th. Has gone on improving steadily, passed nearly all his urine by the penis, and is generally in most excellent condition. On the last day of the month, I discharged him perfectly well. He had to pass his urine more frequently than one in health, but the bladder was fully under his control.

The stone weighed five hundred and nine grains; an inch and a half in its longest diameter, nearly cubical, with rounded angles; surface rough, with mammillary projections, and studded with fine semi-transparent crysals of oxalate of lime; the projections composed of concentric coats of light greenish ox. lime and ox. iron (? the latter appearing in brown highly polished incrustations, which show themselves when the greyish coat is denuded.

